# Research on a Power Management System for Thermoelectric Generators to Drive Wireless Sensors on a Spindle Unit

**DOI:** 10.3390/s140712701

**Published:** 2014-07-16

**Authors:** Sheng Li, Xinhua Yao, Jianzhong Fu

**Affiliations:** 1 School of Mechanical Engineering, Hangzhou Dianzi University, Hangzhou 310018, China; 2 The State Key Lab of Fluid Power Transmission and Control, Zhejiang University, Hangzhou 310027, China; E-Mails: yaoxinhuazju@gmail.com (X.Y.;; fjz@zju.edu.cn (J.F.)

**Keywords:** power management system, thermoelectric generator, wireless sensor, spindle

## Abstract

Thermoelectric energy harvesting is emerging as a promising alternative energy source to drive wireless sensors in mechanical systems. Typically, the waste heat from spindle units in machine tools creates potential for thermoelectric generation. However, the problem of low and fluctuant ambient temperature differences in spindle units limits the application of thermoelectric generation to drive a wireless sensor. This study is devoted to presenting a transformer-based power management system and its associated control strategy to make the wireless sensor work stably at different speeds of the spindle. The charging/discharging time of capacitors is optimized through this energy-harvesting strategy. A rotating spindle platform is set up to test the performance of the power management system at different speeds. The experimental results show that a longer sampling cycle time will increase the stability of the wireless sensor. The experiments also prove that utilizing the optimal time can make the power management system work more effectively compared with other systems using the same sample cycle.

## 1. Introduction

Wireless sensors, engineered as miniaturized, low-cost embedded devices with integrated sensing, processing, and radio communication capabilities, have recently attracted a great deal of attention for use in different monitoring applications [1–4]. Spindle units of machine tools, built within totally closed metallic shells, represent structures of crucial monitoring importance. Though using wireless sensors is a good solution for spindle monitoring, ongoing challenges still exist on how to handle the sensors' power supply. Because of the difficulty in opening the spindle shell and replacing the battery within, the idea of using battery power for wireless sensors has to be rejected. Consequently, in order to create wireless sensors working with long-lasting power supplies, devices that harvest energy from their surroundings are being developed as power sources for wireless sensors [5–7].

Several kinds of ambient energy, such as temperature gradients, mechanical vibrations, radiofrequency waves, and even microbial fuel, have now been tested for conversion into electrical energy [1–4,8,9]. Thermal energy is ubiquitous and can be found in almost any environment. Thermoelectric energy harvesting has been successfully developed for decades for power generation by using waste heat from industrial processes, vehicle exhaust, and space travel [10,11]. The waste heat from spindle units during machining also creates obvious potential for thermoelectric energy harvesting. Moreover, the thermoelectric generator (TEG) can work well without any mechanical motion, so it won't affect the dynamic balancing performance of the spindle. Though thermoelectric generation looks like an ideal source to power wireless sensors in spindle units, there are still hurdles remaining before this application can be adopted. Compared with the application systems described above, which involve heat flows on the kilowatt scale and temperatures of hundreds of degrees Celsius, the temperature change in spindles is relatively small and unstable because it is greatly affected by the working situation of the spindle. These challenging conditions may induce low and fluctuating power density from the output of TEGs. Thus, an additional power management system is needed to help the TEGs output be consistent and also regulate the voltage for wireless sensors.

Power management systems are always used to harvest various low-power energies, such as Microbial Fuel Cells (MFCs), small solar cells, or vibration and thermal energy generators. It is typically designed to boost a low-voltage input to a high-voltage output (e.g., 3.3 V) to continuously or intermittently drive commercial electronic devices. Most existing commercial DC/DC converters are not suitable for low-power energy sources, such as TEGs being used in a spindle. Rechargeable batteries and capacitors are used as part of an energy harvesting system [12–15]. Compared with a capacitor, a rechargeable battery has a higher energy density but requires a significantly longer charging time [13]. A capacitor is more suitable for applications needing power intermittently for only fractions of a second to several minutes [16], which is the case with typical wireless sensors. In addition, capacitors have a longer lifetime than that of rechargeable batteries [13]. As a result capacitors have become more widely used in energy harvesting systems.

When capacitors are used as energy storage devices, the output power and charging/discharging cycles of the power management systems are affected by the capacity of the capacitors [17]. Consequently, many researches focus on optimization of charging/discharging capacitors, and the charging/discharging cycles are always determined by the charging/discharging voltage [17,18]. However, the output of TEGs on spindle bearings is determined by the working situation, and the charging/discharging voltage method is not suitable for this type of application. Most such voltage methods require additional circuits or devices to manage the charging process, which is not feasible for spindle-bearing TEGs due their low power output. Our approach in this paper is to design a transformer-based power management system for harvesting energy from a spindle bearing using thermoelectric generators with charging/discharging capacitors through using the time method. The rest of this paper is organized as follows: Section 2 illustrates the output characteristics of TEGs and the principle of the power management system. Section 3 presents experimental implementation and evaluation of the power management system. The paper concludes in Section 4.

## 2. Power Management System Design and Optimization

### 2.1. Evaluation of the Characteristics of the Thermoelectric Generator with a Spindle Bearing

**Figure 1.**
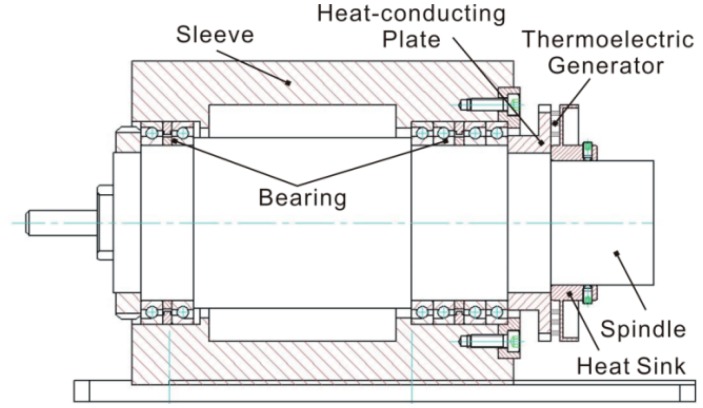
Structure of thermoelectric generator on bearing.

The structure of a thermoelectric generator with a bearing is shown in Figure 1. The heat flow from the spindle bearing is transferred to the thermoelectric generator by a heat-conducting plate, and the generator is mounted on the surface of heat-conducting plate.

The structure includes thermal and electrical characteristics. Based on this feature, all thermal processes are described in electrical terms using the well-known analogies between electrical and thermal domains, presented in Table 1. According to these analogies, the thermo-electrical behavior of a thermoelectric generator with a bearing can be viewed as an electrical network composed of electrical current sources, voltage sources, resistors and capacitors [19].

**Figure 2.**
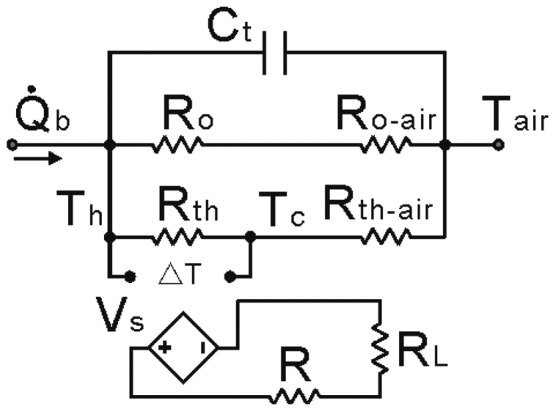
Model of thermoelectric generator with a bearing.

The structure of the bearing includes the shaft, bearing and heat-conducting plate. According to the theory of a thermal network, an equivalent circuit can be built. Combining the thermoelectric generator model, the completed model is shown in Figure 2 [20]. In this model, the output voltage of the thermoelectric generator *V_s_* is viewed as a dependent voltage source, and *V_s_* can be expressed as follows:

(1) $$V_{s}=S\left(T_{h}-T_{c}\right)$$

where *S* is the Seebeck coefficient of the thermocouple, *T_h_* and *T_c_* are the temperatures at the hot side and cold side respectively. *Q̇_b_*. is the heat flow of the bearing, and it can be expressed as [21]:

(2) $${\dot{Q}}_{b}=1.047\times 10^{-4}Mn$$

where *M* is the friction moment of the bearing, and *n* is the rotation speed of the spindle. The units for *M* and *n* are N·mm and r/min, respectively. *T_air_* is the environmental temperature of the air. *R_th_* is the thermal resistance of the thermoelectric generator, and *R_o_* is the thermal resistance of the metal. *C_t_* is the entire heat capacity of the bearing structure. The thermal resistance between the air and the metal is *R_o-air_*, and the thermal resistance between the air and the generator is *R_th-air_*. *R* is the internal resistance of the generator, and *R_L_* is the load resistance.

According to this model, the entire structure can be viewed as a power source, and the power depends on the input heat flow *Q̇_b_*. The model can be expressed as:

(3) $$\{\begin{array}{c} {\dot{Q}}_{C_{t}}=C_{t}\frac{dT_{C_{t}}\left(t\right)}{dt}\\ T_{C_{t}}\left(t\right)=T_{h}\left(t\right)-T_{\text{air}}\\ T_{h}\left(t\right)-T_{\text{air}}={\dot{Q}}_{o}\left(R_{o}+R_{o-\text{air}}\right)\\ T_{h}\left(t\right)-T_{\text{air}}={\dot{Q}}_{th}\left(R_{th}+R_{th-\text{air}}\right)\\ {\dot{Q}}_{C_{t}}+{\dot{Q}}_{o}+{\dot{Q}}_{th}={\dot{Q}}_{b}\\ \overset{\left[T_{h}\left(t\right)-T_{c}\left(t\right)\right]}{\;}/\underset{R_{th}}{\;}=\overset{\left[T_{c}\left(t\right)-T_{\text{air}}\right]}{\;}/\underset{R_{th-\text{air}}}{\;} \end{array}$$

where *Q̇_ct_*, *Q̇_o_* and *Q̇_th_* are the heat flow which goes through *C_t_*, *R_o_* and *R_th_*, respectively. *T_c_* (*t*) is the temperature difference between the two sides of the heat capacity, *T_h_* (*t*) is the temperature of the TEG hot side, and *T_c_* (*t*) is the temperature of the TEG cold side. *T_c_* (*t*), *T_h_* (*t*) and *T_c_* (*t*) are all functions of time (*t*). When the time (*t*) is equal to 0, all the temperatures are *T_air_*. The variables, which depend on the geometric structure of the spindle, are given in Table 2.

**Figure 3.**
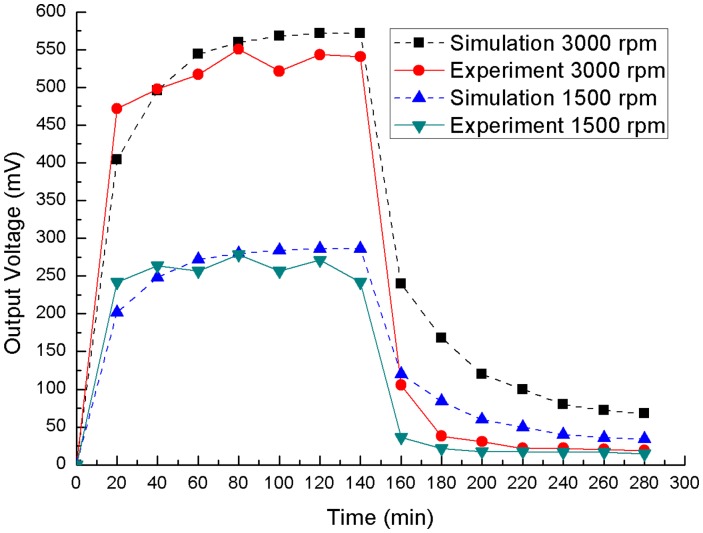
Output voltage of simulation and experiment.

According to this structure and Equation (3), the relationship between the output voltage and time can be simulated in software SPICE, and the simulation is shown in Figure 3. The spindle rotates from 0 min to 140 min, and the speeds are 1500 rpm and 3000 rpm. The experimental output voltage, which is measured under load, is also shown in Figure 3, and the simulation and experiment tests show the same change trends. It can be shown that the voltage usually fluctuates at different speeds, and it is the main reason to charge and discharge the capacitance by time not by voltage. In the experiment, *T_air_* is 293 K, the maximum *T_h_* is about 326 K and the maximum *T_c_* is about 304 K.

### 2.2. Transformer-Based Power Management System

**Figure 4.**
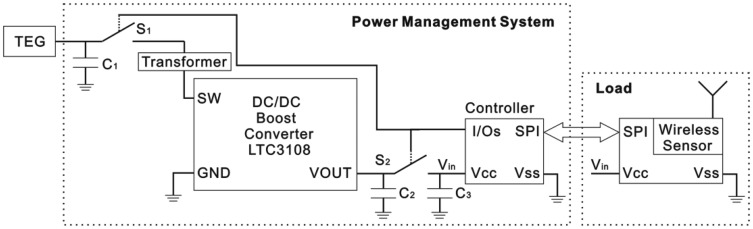
Schematic diagram of power management system.

Different power management systems for TEG power harvesting have been studied for intermittent load driving applications such as intermittent wireless transmission of sensed information. A transformer-based power management system, including two super capacitors, is used to harvest microbial fuel cell (MFC) power [22]. Two switches are used in this power management system, and the switches are controlled based on the voltage of the super-capacitors. Unlike the MFC, the TEG, mounted on the heat-conducting plate, cannot supply stable voltage, as shown in Figure 3, so the switches control method is not suitable for the TEG in a spindle unit. A simple improvement is illustrated in this paper, and the power management system is shown in Figure 4. According to this bearing working situation, two switches and a load are operated by a controller. The power management system includes three super-capacitors: the first one is connected to the TEG, the second one is placed after the DC/DC converter, and the last one is installed before the controller. The first super-capacitor (*C_1_*) accumulates energy from the TEG and drives the following DC/DC converter. The second super-capacitor (*C_2_*), whose capacitance must be confirmed by the target load, saves the power from the converter and drives the load. The last super-capacitor (*C_3_*) provides power for the controller, which operates two switches and the load through two input/output (I/O) pins and a serial peripheral interface (SPI) bus, respectively. The first switch (*S_1_*) determents the charging and discharging time of the first super-capacitor (*C_1_*), and the second switch (*S_2_*) controls the charging and discharging time of the second (*C_2_*) and third (*C_3_*) super-capacitors.

### 2.3. Control Strategy for TEGs on Bearing

**Figure 5.**
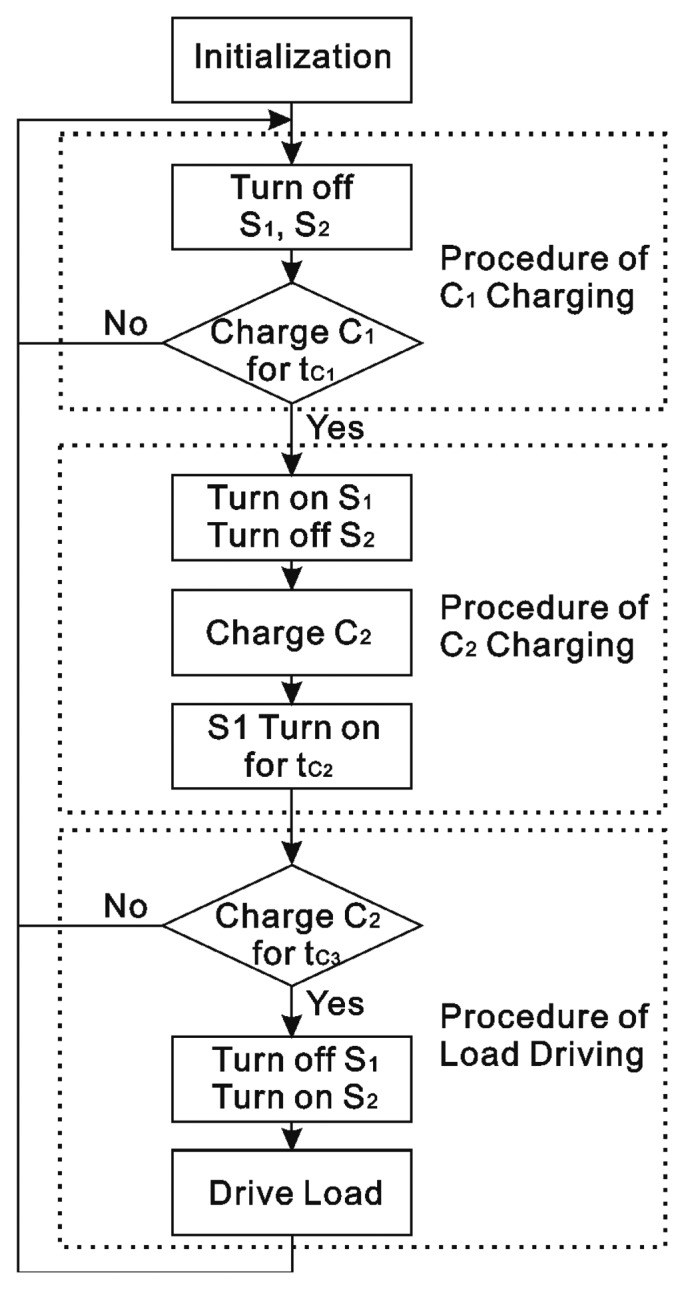
Control strategy for TEGs on bearing.

The control strategy is shown in Figure 5, and it includes four procedures which are initialization, *C_1_* charging, and *C_2_* charging and load driving. In the initialization procedure, the bearing starts to rotate. In the *C_1_* charging procedure, the switches *S_1_* and *S_2_* must be turned off and the charging time *t_c_*_1_ is dependent on the internal resistance of TEGs. In the *C_2_* charging procedure, *S_1_* is turned on for *t*_*c*_2__. After that, if *C_2_* charges for *t*_*c*_3__, which is also the operation period time, the procedure of load driving begins.

**Figure 6.**
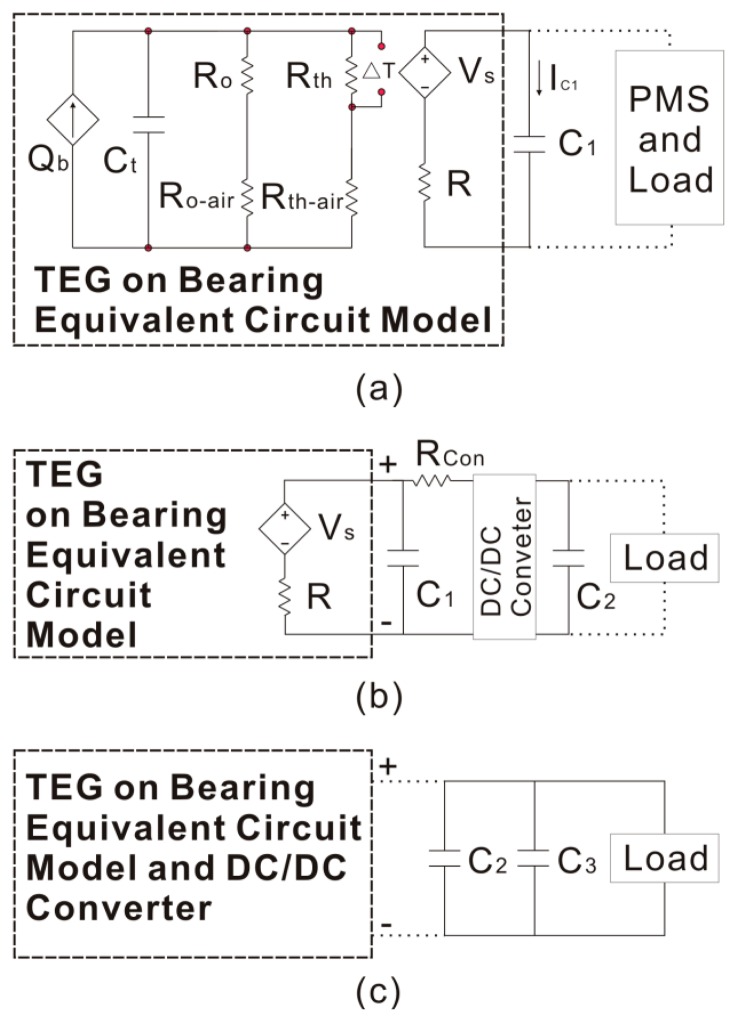
Equivalent circuit (**a**) *C_1_* charging; (**b**) *C_2_* charging; (**c**) load driving.

Equivalent circuit model for TEG on bearing and the overall circuit schematic during *C_1_* charging is shown in Figure 6a.

The circuit, containing *V_s_*, *C_1_* and *R*, can be expressed as:

(4) $$\{\begin{array}{c} I_{C_{1}}=C_{1}\frac{dU_{C_{1}}\left(t\right)}{dt}\\ U_{C_{1}}\left(t\right)=V_{s}-RI_{C_{1}} \end{array}$$

where *I*_*C*_1__ is the current on the electric capacity *C_1_*, and *U*_*C*_1__(*t*) is the voltage of the electric capacity *C_1_*. According to Equation (4) *U*_*C*_1__(*t*) can be written as follows:

(5) $$U_{C_{1}}\left(t\right)=V_{s}-\Delta Ve^{-\frac{t}{RC_{1}}}$$

where Δ*V* is the difference between *V_s_* and the initial voltage *U*_*C*_1__(*t*_0_). The average power (*P_a_*) stored in the electric capacity *C_1_* during the *C_1_* charging can be expressed as:

(6) $$P_{a}=\frac{1}{2t_{C_{1}}}C_{1}\left\{{\left[U_{C_{1}}\left(t_{C_{1}}\right)\right]}^{2}-{\left[U_{C_{1}}\left(t_{0}\right)\right]}^{2}\right\}$$

Based on Equations (5) and (6) this can be altered as follows:

(7) $$P_{a}\left(a,b\right)=\frac{V_{s}^{2}R}{2b}\left[{\left(1-ae^{-b}\right)}^{2}-{\left(1-a\right)}^{2}\right]$$

where Δ*V* = *aV_s_* and *b* = *t_C_*_1_/*RC*_1_ (0 < *a* < 1, *b* > 0). Figure 3 shows that *V_s_* is unstable, so the maximum value of *P_a_* should be found at a different *V_s_*. According to Equation (7), the maximum value of *P_a_* only depends on the value of *a* and *b*, so that the value of *a* and *b* is the key to finding the maximum value at a different *V_s_*. The maximum value of the average power can be found through MATLAB, and it occurs at *a* = 0.53 and *b* = 0.08 (Δ*V* = 0.53*V_s_* and *t_C_*_1_ = 0.08*RC*_1_). The optimum values of *C_1_* charging time *t*_*C*_1__, the initial voltage *U*_*C*_1__(*t*_0_) and the final charge voltage *U*_*C*_1__(*t_C_*_1_) are 0.08*RC*_1_, 0.47*V_s_* and 0.51*V_s_*, respectively. In Figure 6b, the equivalent circuit model for TEG and the overall circuit schematic during *C_2_* charging is shown. According to this circuit, the relationship between the initial voltage *U*_*C*_1__(*t*_0_) and the final charge voltage *U*_*C*_1__(*t*_*C*_1__) can be expressed as:

(8) $$U_{C_{1}}\left(t_{0}\right)=\frac{R_{\text{Con}}}{R+R_{\text{Con}}}V_{s}-\left(\frac{R_{\text{Con}}}{R+R_{\text{Con}}}V_{s}-U_{C_{1}}\left(t_{C_{1}}\right)\right)e^{-\frac{t_{C_{2}}}{\frac{RR_{\text{Con}}}{R+R_{\text{Con}}}C_{1}}}$$

where *R_Con_* is the equivalent resistance of the DC/DC converter. According to the value of the initial voltage *U*_*C*_1__(*t*_0_) and the final charge voltage *U*_*C*_1__(*t*_*C*_1__), the *C_2_* charging time *t*_*C*_2__ can be determined. The equivalent circuit of the load driving procedure is shown in Figure 6c, and the energy changing on *C_2_* and *C_3_* can be expressed as:

(9) $$\{\begin{array}{c} \Delta E_{2}=\frac{C_{2}}{2}\left({U_{C_{2}C}}^{2}-{U_{C_{2}D}}^{2}\right)\\ \Delta E_{3}=\frac{C_{3}}{2}\left({U_{C_{3}C}}^{2}-{U_{C_{3}D}}^{2}\right)\\ \Delta E_{3}=P_{\text{Controller}}t_{C_{3}}+E_{\text{Load}}\\ \Delta E_{2}=\Delta E_{3}\\ U_{C_{2}D}=U_{C_{3}C}\\ U_{C_{3}D}>U_{\text{Drive}}\\ U_{C_{2}C}<U_{\text{Max}\_\text{Output}} \end{array}$$

where Δ*E*_2_ and Δ*E*_3_ are the energy changing on *C_2_* and *C_3_*, *U*_*C*_2__*_C_* and *U*_*C*_2__*_D_* are the voltage of *C_2_* after charging and discharging, *U*_*C*_3__*_C_* and *U*_*C*_3__*_D_* are the voltage of *C_3_* after charging and discharging, *P_Controller_* is the power of the controller, *t*_*C*_3__ is the period time, *E_Load_* is the energy consumed by the load in one period, *U_Drive_* is the minimum drive voltage, *U_Max_Output_* is the maximum output voltage of LTC3108 (Linear Technology Corporation, Milpitas, CA, USA). Δ*E*_2_ is consumed by charging *C_3_*, and Δ*E*_3_ is consumed by the controller and load. Because the load runs one time in one period, *E_Load_* is a constant. *t*_*C*_3__ is dependent on the frequency of the sending data, and Equation (9) is the conditions of a normal run. When *V_s_* fluctuates during energy harvesting, *U*_*C*_2__*_C_* and *U*_*C*_3__*_D_* will change. If *U*_*C*_3__*_D_* meets the condition of *U*_*C*_3__*_D_*>*U_Drive_*, the power management system can work stably.

## 3. Simulation and Experimental Evaluation of the Proposed Power Management System

**Figure 7.**
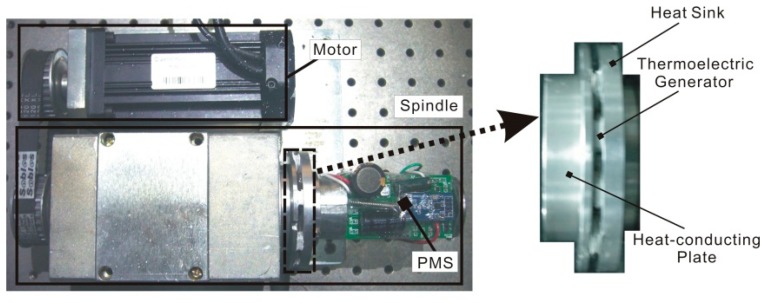
Experimental platform.

An experimental platform is set up with TEGs, including nine thermoelectric devices, around the spindle, and the volume of the whole platform is about 200 × 200 × 100 mm^3^ as shown in Figure 7. The TEGs are thermally in parallel but electrically in series. A DC motor (Syntron Company, Beijing, China) is used to control the rotation speed of the spindle through synchronous wheels with 300 W rated power output. The heat flow from the spindle bearing is transferred to the TEGs by a heat-conducting plate. The TEG Xinghe Semiconductor Company, Jiangsu, China; chosen for this study is 8 × 8 × 2.5 mm^3^ in size with a 2.8 mV/K Seebeck coefficient. Its internal resistance is 3 Ω, and its thermal resistance is 32.6 W/K. The weight of the power management system and the TEGs is approximately 60 g.

**Figure 8.**
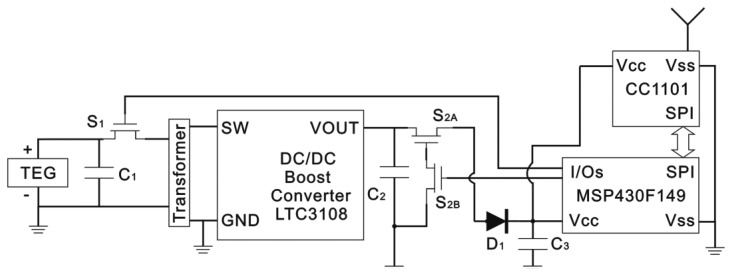
Circuit of power management system.

As shown in Figure 8, the controller is a MSP430F149 (Texas Instruments, Dallas, TX, USA) and its low-power mode 3 is used in this experiment. The wireless module is a CC1101 (Texas Instruments). *S*_1_ is a 1.5 V drive N-channel MOSFET, and its type is a RQ1C075UN (ROHM, Kyoto, Japan). *S*_2_, shown in Figure 4b, is composed of *S*_2A_ and *S*_2B_ · *S*_2A_ is a 1.5 V drive P-channel MOSFET, and its type is a RQ1A070ZP (ROHM). *S*_2B_ is the same as *S*_1_ · *D*_1_ is used to keep *C*_2_ charging *C*_3_, and its type is a 1N4007, which has about a 0.5 V voltage drop in the experiment.

Before the power management system experiment, some unknown variables should be determined, and they are shown in Table 3. For offering enough response time for *S*_1_, choose *C*_1_ to 1 F. *C*_2_ and *C*_3_ is dependent on the load, and they are 1500 μF and 4700 μF, respectively. *R*, which depends on the thermoelectric device, is equal to 27 Ω. *R_Con_*, *P_Controller_* and *E_Load_* can be obtained by the measurement. Because of the voltage drop on *D*_1_, *U_Max_Output_* is set to 4.1 V.

**Figure 9.**
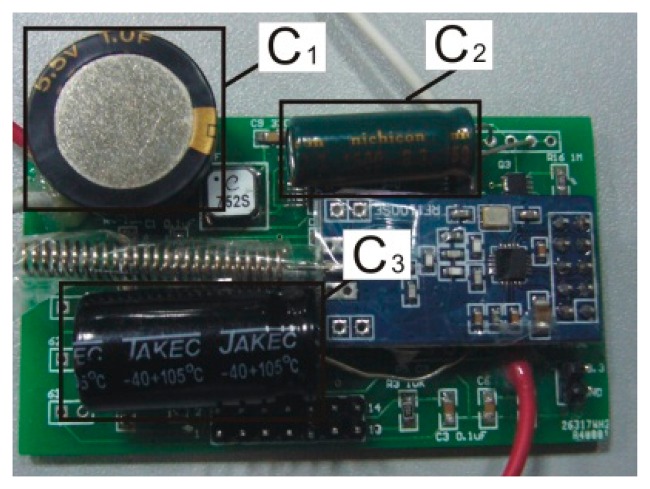
Photo of power management system.

After obtaining those variables, the controlling time can be determined. According to Equation (7), the optimal *t*_*C*_1__ is 2 s. After that, *t*_*C*_2__ can be calculated through Equation (8), and it is 13 s. *t*_*C*_3__ is dependent on the time cycles (*T_Data_*) of the sending data, and *T_Data_* = *t*_*C*_3__ 100 s. Finally, the power management system can be used in this experiment according to those three times, and the photo of the power management system is shown in Figure 9.

**Figure 10.**
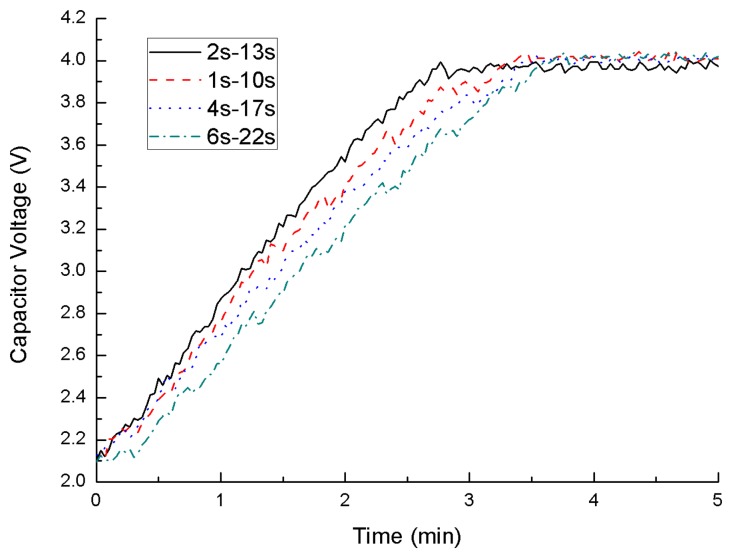
Capacitor voltages of *C*_2_.

Before putting the power management system on the spindle, a test, using different *t*_*C*_1__ to charge *C*_2_ from 2.1 V to 4.1 V with 100 mV input, is performed. *t*_*C*_1__ is equal to 2 (the optimal value), 1, 4 and 6 s. After that, *t*_*C*_2__ can be calculated through Equation (8), and they are 13, 10, 17 and 22 s. The charging mode, using the optimal value, has the higher efficiency than the others as shown in Figure 10. The optimal charging mode can offer more power than others.

**Figure 11.**
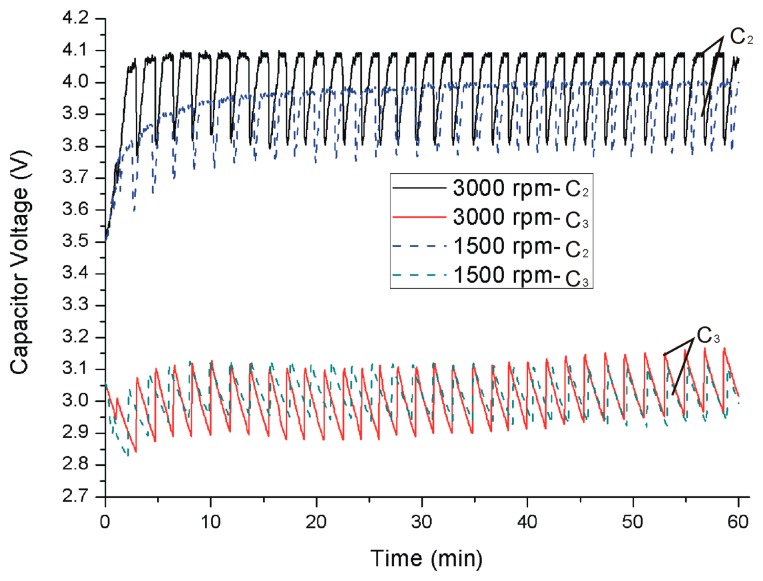
Capacitor voltages of *C*_2_ and *C*_3_.

After the test, the power management system is installed on the spindle with the optimal charging set. In the experiment, the spindle rotates at 1500 rpm and 3000 rpm. The voltages of *C*_2_ and *C*_3_ are shown in Figure 11. At the beginning *C*_2_ and *C*_3_ are charged to 3.5 V and 3 V respectively, and then the spindle will start rotating. At 3000 rpm, the voltage of *C*_2_ jumps to 4.1 V and fluctuates between 3.8 V and 4.1 V. At 1500 rpm, the voltage of *C*_2_ rises to 3.8 V and fluctuates between 3.6 and 3.9 V, and the voltage of *C*_2_ also has a rising trend. This phenomenon is caused by the different speeds. As shown in Figure 3, the higher speed can produce higher output voltage, so the voltage of *C*_2_ can reach to 4.1 V at 3000 rpm.

**Figure 12.**
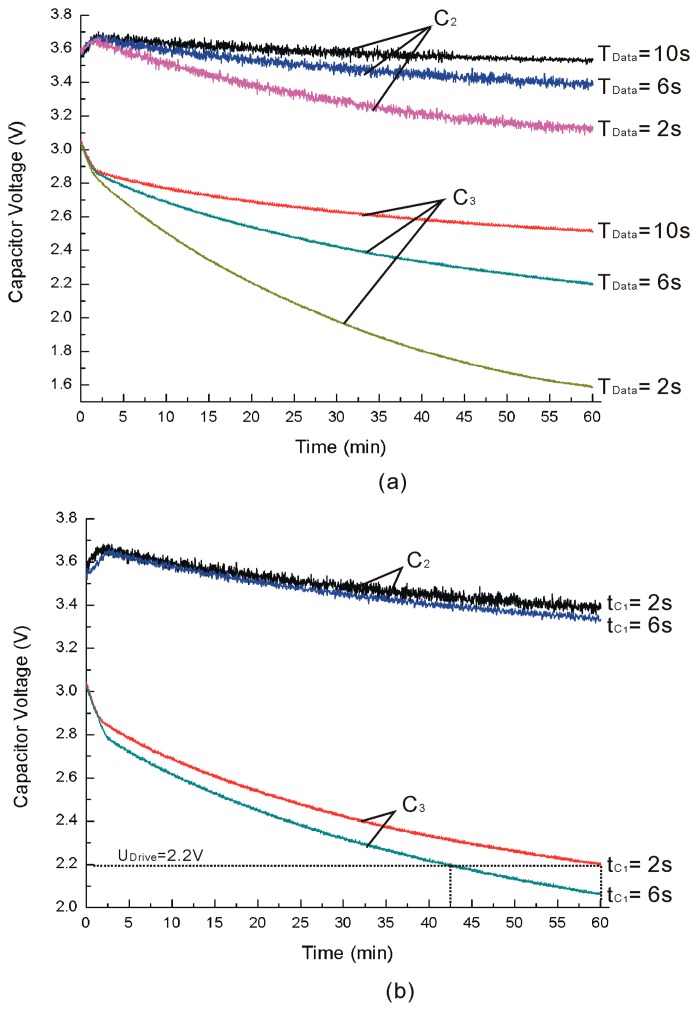
Capacitor voltages of *C*_2_ and *C*_3_. (**a**) Different time cycles; (**b**) Different *t*_*C*_1__.

For analyzing the power management system working at different sampling cycles of the wireless sensor, the time cycles (*T_Data_*), which are equal to *t*_*C*_3__, are set as 2, 6 and 10 s with *t*_*C*_1__ equal to 2 s and rotating at 1500 rpm, and the voltages of *C*_2_ and *C*_3_ are shown in Figure 12a. Because *T_Data_* is small, the voltages of *C*_2_ and *C*_3_ do not fluctuate like those shown in Figure 11. It is clear that *T_Data_* is so small the voltages of *C*_2_ and *C*_3_ cannot be held on a stable voltage, and the voltages decrease quickly with *T_Data_* reducing. This phenomenon can also be explained by Equation (9). With *T_Data_* reducing, the power consumption increases and Equation (9) cannot be satisfied. Different *t*_*C*_1__ with *T_Data_* equal to 6 s are shown in Figure 12b. The voltages of *C*_2_ and *C*_3_ using the optimal *t*_*C*_1__ decrease slower than those not using the optimal value, and it is the same as shown in Figure 11. It is clear that the voltages of *C*_3_ will be below 2.2 V (*U_Drive_*) with *t*_*C*_1__=6 s and the wireless sensor cannot work stably under such low voltage settings. Using the optimal *t*_*C*_1__=2 s, the voltages of *C*_3_ can be kept higher than *U_Drive_* for 60 min. On the other hand, the voltages of *C*_3_ can be kept higher than *U_Drive_* for 42.5 min when using *t*_*C*_1__=6 s and *t*_*C*_2__=17 s. That is to say, the optimal *t*_*C*_1__ can increase by a 41.2% stable working time when compared with *t*_*C*_1__=6 s. Similar results are measured for other comparative experiments between *t*_*C*_1__=2 s and other values. As the sampling cycle time of the wireless sensor is longer, the wireless sensor works more stably.

## 4. Conclusions

While the power generating capacity of TEGs can be improved in terms of using different thermoelectric materials and/or choosing different heat exchange structures, a power management system is always essential to enhance the energy harvesting and usage efficiency. This study presents a transformer-based power management system and its control strategy is to make the wireless sensor work stably at different speeds. Because the output of the TEG varies with the speed of the spindle, the strategy uses charging/discharging time of the capacitors instead of the voltage to control energy harvesting. The proposed power management system is installed on a spindle to test its working state at different speeds. The experiments show that the optimal time can offer more power than other times, and it is also found that the power management system can drive the wireless sensor very well when the sampling cycle time of the sensor is relatively long. The results also show that the small sampling cycle time (*T_Data_*) will induce the power management system to work unstably. However, the optimal *t*_*C*_1__ of the power management system still improves the performance of TEG under several-seconds during sampling cycles and increases to a 41.2% stable working duration compared with *t*_*C*_1__=6 s under *T_Data_* = 6 s. The findings prove that the optimal time shown in this paper can make the power management system have longer work times when compared with other systems during the same cycle times.

## Figures and Tables

**Table 1.** Analogies between thermal and electrical variables.

**Thermal Variable**	**Electrical Variable**
Heat flow *Q* (W)	Current flow I (A)
Temperature T (K)	Voltage V (V)
Thermal resistance R_th_ (KW^−1^)	Electrical resistance R (Ω)
Thermal mass C_th_ (JK^−1^)	Electrical capacity C (F)

**Table 2.** Variables used in the model of a thermoelectric generator with a bearing.

**Variable**	**Value**	**Unit**
*R_th_*	3.62	K/W
*R_th-air_*	3.19	K/W
*R_o_* + *R_th-air_*	0.14	K/W
*_R_*	27	Ω
*R_L_*	1000	Ω
*C_t_*	6870	J/K
*Q̇_b_*	301.22	W

**Table 3.** Variables used in power management system experiment.

**Variable**	**Value**	**Unit**
*C*_1_	1	F
*C*_2_	1500	μF
*C*_3_	4700	μF
*_R_*	27	Ω
*R_Con_*	22	Ω
*P_Controllar_*	50	μW
*E_Load_*	0.2	mJ
*U_Drive_*	2.2	V
*_UMax_Output_*	4.1	V
