# Correction: Li, S. *et al.* Research on a Power Management System for Thermoelectric Generators to Drive Wireless Sensors on a Spindle Unit. *Sensors* 2014, *14*, 12701–12714

**DOI:** 10.3390/s150820541

**Published:** 2015-08-20

**Authors:** Sheng Li, Xinhua Yao, Jianzhong Fu

**Affiliations:** 1 The State Key Laboratory of Fluid Power Transmission and Control, Zhejiang University, Hangzhou 310027, China; E-Mails: yaoxinhuazju@gmail.com (X.Y.); fjz@zju.edu.cn (J.F.); 2 School of Mechanical Engineering, Hangzhou Dianzi University, Hangzhou 310018, China

The authors wish to make the following corrections to this paper [1]. The authorship should be “Sheng Li ^1,2,^*, Xinhua Yao ^1^ and Jianzhong Fu ^1^”, and the affiliations should be changed from:

^1^  School of Mechanical Engineering, Hangzhou Dianzi University, Hangzhou 310018, China
^2^  The State Key Lab of Fluid Power Transmission and Control, Zhejiang University, Hangzhou 310027, China; E-Mails: yaoxinhuazju@gmail.com (X.Y.); fjz@zju.edu.cn (J.F.)

to:

^1^  The State Key Laboratory of Fluid Power Transmission and Control, Zhejiang University, Hangzhou 310027, China; E-Mails: yaoxinhuazju@gmail.com (X.Y.); fjz@zju.edu.cn (J.F.)
^2^  School of Mechanical Engineering, Hangzhou Dianzi University, Hangzhou 310018, China

Also, the Acknowledgments section should be changed from:

“This work is supported by the National Natural Science Foundation of China (No. 51105336) and the National Natural Science Foundation of China (No. 61100101).”

to:

“This work is supported by the National Natural Science Foundation of China (No. 51105336), the National Natural Science Foundation of China (No. 61100101) and the Fundamental Research Funds for the Central Universities.”

The authors would like to apologize for any inconvenience caused to the readers by these changes.
